# Quantitative Proteomic Analysis of Lysine Malonylation in Response to Salicylic Acid in the Roots of *Platycodon grandiflorus*

**DOI:** 10.3390/ijms26031392

**Published:** 2025-02-06

**Authors:** Wanyue Ding, Yingying Duan, Yuqing Wang, Jizhou Fan, Weiyi Rao, Shihai Xing

**Affiliations:** 1 College of Pharmacy, Anhui University of Chinese Medicine, Hefei 230012, China; dwy111@stu.ahtcm.edu.cn (W.D.); dyy@stu.ahtcm.edu.cn (Y.D.); 19991122wyq@stu.ahtcm.edu.cn (Y.W.); 17805652827@stu.ahtcm.edu.cn (J.F.); r13694358rwy@163.com (W.R.); 2 Institute of Traditional Chinese Medicine Resources Protection and Development, Anhui Academy of Chinese Medicine, Hefei 230012, China; 3 MOE—Anhui Joint Collaborative Innovation Center for Quality Improvement of Anhui Genuine Chinese Medicinal Materials, Hefei 230038, China

**Keywords:** *Platycodon grandiflorus*, salicylic acid, lysine malonylation, medicinal plants, qualitative proteome, post-translational modification

## Abstract

Salicylic acid, as a plant hormone, significantly affects the physiological and biochemical indexes of soluble sugar, malondialdehyde content, peroxidase, and superoxide dismutase enzyme activity in *Platycodon grandiflorus*. Lysine malonylation is a post-translational modification that involves various cellular functions in plants, though it is rarely studied, especially in medicinal plants. In this study, the aim was to perform a comparative quantitative proteomic study of malonylation modification on *P. grandiflorus* root proteins after salicylic acid treatment using Western blot with specific antibodies, affinity enrichment and LC-MS/MS analysis methods. The analysis identified 1907 malonyl sites for 809 proteins, with 414 proteins and 798 modification sites quantified with high confidence. Post-treatment, 361 proteins were upregulated, and 310 were downregulated. Bioinformatics analysis revealed that malonylation in *P. grandiflorus* is primarily involved in photosynthesis and carbon metabolism. Physiological and biochemical analysis showed that salicylic acid treatment increased the malondialdehyde levels, soluble protein, superoxide dismutase, and peroxidase activity but did not significantly affect the total saponins content in *P. grandiflorus*. These findings provide an important basis for exploring the molecular mechanisms of *P. grandiflorus* following salicylic acid treatment and enhance understanding of the biological function of protein lysine malonylation in plants.

## 1. Introduction

*Platycodon grandiflorus* (Jacq.) A. DC is a medicinal and perennial herbaceous plant with ornamental flowers and edible roots that is predominantly distributed in China, South Korea, Japan, Russia, and other places [1]. Its medicinal use dates back over 2000 years, first recorded in the Shennong classic of *Materia Medica* [2]. *P. grandiflorus* is rich in bioactive compounds, including saponins, flavonoids, phenols, polysaccharides, volatile oils, and amino acids, with triterpene saponins being the primary active [3], most abundant component in its root [4]. *P. grandiflorus* has notable pharmacological properties, including antioxidant, immunostimulatory, anti-inflammatory, antitumor, anti-obesity, and anti-depressant effects [5]. According to the 2020 edition of the *Pharmacopoeia of the People’s Republic of China*, *P. grandiflorus* is used for promoting the health of the lungs, soothing the throat, eliminating phlegm, and discharging pus, making it effective for treating coughs, chest congestion, sore throat, pulmonary carbuncles, and pus [1].

**Figure 1:**
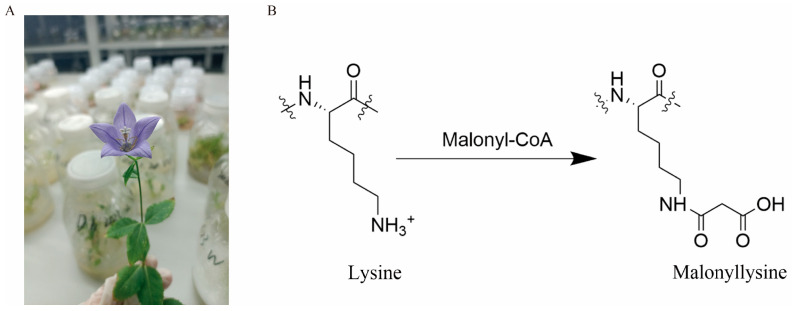
*P. grandiflorus* and malonyl-lysine formation. (**A**) The plant of *P. grandiflorus*. (**B**) The process of protein lysine malonylation.

Post-translational protein modification (PTM), a covalent event occurring post-DNA transcription and translation, plays a pivotal role in regulating cellular processes [6]. PTMs predominantly target amino acids such as lysine, serine, threonine, tyrosine, and histidine. As molecular biology advances, PTM is becoming more popular, among which lysine malonylation (Kmal) has garnered increasing attention since its discovery (Figure 1B) [7]. Kmal is prevalent in mammalian and bacterial cells and causes more significant structural changes than lysine acetylation and methylation [8]. Chemically, the acidic modification adds a larger acyl group to lysine (malonylation), shifting its charge from +1 to −1, which can significantly alter the protein structure and function [9,10]. Studies have shown that malonylation modification occurs in the histones of various prokaryotes and eukaryotes, including *Saccharomyces cerevisiae* [11], *Bacillus amylolyticus* [12], *Saccharopolyspora erythraea* [13], mice [14], and rice [15], including its role in regulating gene transcription. In addition, malonylation has been observed in organelles such as the nucleus and chloroplasts, highlighting its role in various metabolic activities in organisms [16]. However, despite some progress in lysine modification research, malonylation, especially in medicinal plants, remains underexplored, warranting further investigation.

Salicylic acid (SA), a plant-derived phenolic compound and natural hormone [17], regulates various processes of plant growth and development, including thermogenesis [18], flowering induction [19], stress resistance [20], and plant growth [21]. In addition, several studies have shown that SA increases the content of secondary metabolites in plants. For instance, 10 µM SA treatment regulates growth and promotes secondary metabolite production in turmeric [22]. Notably, several studies have emphasized the regulatory role of SA in saponins biosynthesis. In *Conocarpus erectus* L. and *Populus deltoides* L., SA treatment significantly reduced H_2_O_2_ and O_2_^−^-levels and activated antioxidant enzymes such as superoxide dismutase (SOD) and peroxidase (POD) [23]. Spraying 1 mM SA decreases H_2_O_2_, SOD, protein, and malondialdehyde (MDA) levels in *Corylus avellana* L. [24]. MDA, a primary lipid peroxidation product, is a key indicator [25] widely used to gauge membrane damage in plants [26]. SA is an important signaling molecule for plant immunity in response to abiotic stress [27]. To investigate the Kmal level of *P. grandiflorus* treated with exogenous SA, we used immunoaffinity precipitation and high-resolution LC-MS/MS to quantitatively analyze the Kmal of the *P. grandiflorus* proteome.

In this study, we aimed to perform a quantitative proteomic analysis of lysine malonylation in *P. grandiflorus* based on its physiological and biochemical changes after treatment with SA. Our findings provide important insights into plants’ stress response mechanisms and enhance the utilization of medicinal plants, filling a gap in malonylation research for *P. grandiflorus* and paving the way for subsequent research in the area.

## 2. Results

### 2.1. Difference of Biochemical Index and Component Content

**Figure 2:**
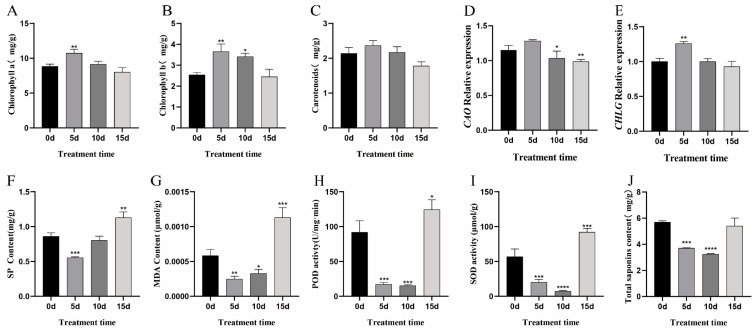
The variation in physiological and biochemical indexes, total saponin content, and gene transcription level by salicylic acid treatment. (**A**) Chlorophyll a content; (**B**) chlorophyll b content; (**C**) the carotenoid content; (**D**) relative expression of *CAO*; (**E**) relative expression of *CHLG* gene; (**F**) the SP content; (**G**) the MDA content; (**H**) the POD activity; (**I**) the SOD activity; (**J**) the total saponin content. The data represent the average value and standard error of three replicates. The asterisks of *, **, ***, and **** above each column show significant differences in *p* ≤ 0.05, *p* ≤ 0.01, *p* ≤ 0.001, and *p* ≤ 0.0001, respectively.

Compared to the control group, the leaves of chlorophyll a, chlorophyll b, and carotenoids in *P. grandiflorus* initially increased and then decreased with SA treatment (Figure 1). The concentrations of these three components peaked around day 5, then declined and dropped below the levels observed in the control group by day 15. Chlorophyllide a oxidase (CAO) and chlorophyll synthase (CHLG) are key catalytic enzymes involved in chlorophyll synthesis. CAO catalyzes chlorophyllide b synthesis, while CHLG catalyzes chlorophyll a and b biosynthesis from chlorophyllide a and b [28]. Under SA treatment, *CAO* and *CHLG* gene expression initially increased and then decreased, which was consistent with the change in chlorophyll levels (Figure 2D,E).

The soluble protein (SP) levels were similar to that of MDA, both showing a decrease followed by an increase during the treatment. A slight increase was observed on day 5, and the rate of increase became significantly higher after day 10, with the highest level observed on day 15. The POD and SOD activities followed a similar pattern, initially decreasing and then increasing. In contrast to SP and MDA, both POD and SOD activities showed an overall decrease during the first 10 days of treatment. However, by day 15, their activities increased sharply, with SOD activity increasing 1.6-fold and POD activity increasing 1.3-fold compared to day 0. As the treatment progressed, the total saponin content in *P. grandiflorus* also initially decreased and then increased.

### 2.2. Detection of Kmal Modification in P. grandiflorus Under Exogenous SA Treatment

**Figure 3:**
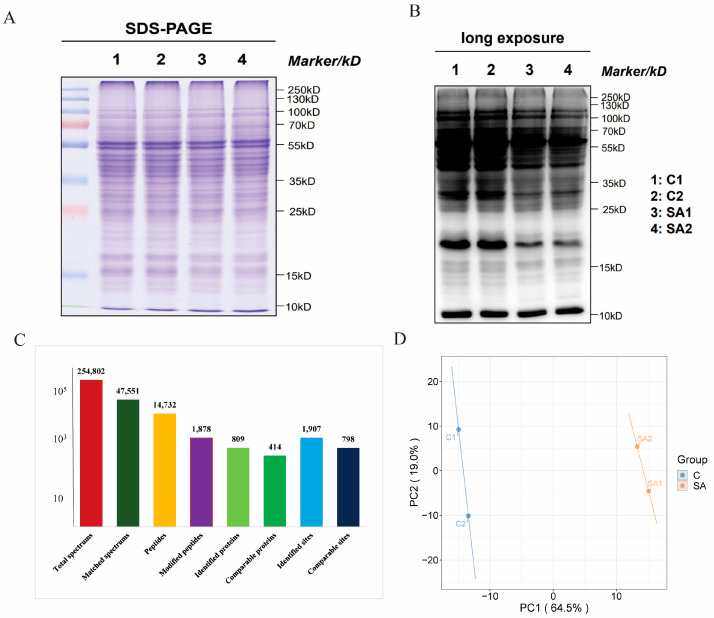
Malonylated protein analysis in *P. grandiflorus*. (**A**) SDS-PAGE of proteins of *P. grandiflorus*, dyed with Coomassie bright blue; (**B**) Western blot image of proteins’ malonylation in *P. grandiflorus*. C1 and C2 are samples of control, SA1 and SA2 are samples treated with SA; the molecular weight of protein is marked on the right; 20 mg of protein was loaded in each sample; (**C**) the overall number of malonyl acyl sites and proteins identified; (**D**) a PCA analysis between controls and treatments. The horizontal and vertical axes show the interpretability of PC1 and PC2. C1 and C2 are samples of control, SA1 and SA2 are samples treated with SA.

Western blotting with a specific antibody was used to detect the Kmal-modified signal in both the control and treatment groups. Sodium dodecyl sulfate–polyacrylamide gel electrophoresis (SDS-PAGE) separation revealed that the proteins in *P. grandiflorus* were primarily distributed within the range of 10–120 kDa, and SA treatment induced certain differences in the proteome (Figure 3A). Western blot analysis showed that Kmal modification was widely present in the proteome of *P. grandiflorus*, predominantly enriched in the 25–75 kDa range. Significant differences in the patterns in the 15–20 kDa and 25–40 kDa regions were observed between control and treatment groups (Figure 3B). This indicates that Kmal modification in the proteome of *P. grandiflorus* may play a role in the response to SA treatment.

### 2.3. Proteome-Wide Identification of Kmal in Roots of P. grandiflorus

To comprehensively analyze Kmal in the proteome of *P. grandiflorus*, we used high-resolution LC-MS/MS to identify the trypsin-digested proteins. Overall, 254,802 spectra were identified, with 47,551 spectra matching the theoretical second-order spectra (Figure 3C). Malonylation was widespread in the *P. grandiflorus* proteome, with 1907 malonylation sites identified across 809 proteins. Among these, 414 proteins and 798 modification sites were confidently quantified. Additionally, we identified 14,732 peptides, with 1878 peptides exhibiting malonylation (Supplementary Table S1). The raw data for all identified malonylated peptides are presented in Supplementary Table S1. In this experiment, 135,399 valid spectra were identified from 895,928 secondary spectra detected using mass spectrometry. The MS/MS spectra of the five peptide segments that were malonylated are shown in Supplementary Figure S1.

Overall, 35,576 peptide sequences were identified, including 33,299 unique peptide sequences, and 5217 proteins were identified, with 3850 proteins able to be quantified by specific peptide sequences (Supplementary Figure S2A). Most of the peptides carried two–three charges and ranged from seven to twenty amino acids, meeting quality control standards (Supplementary Figure S2B). Principal component analysis of the relative quantification values of *P. grandiflorus* proteins revealed that PC1 accounted for 64.5% and PC2 accounted for 19.0% of the variance (Figure 3D). Database search results of mass data can obtain signal intensity values for each peptide segment in each sample, and box plots were used to represent the intensity values of modification sites across different samples (Supplementary Figure S2C) and to explore the distribution and differences in modification site intensity values among samples. The results showed that the mean values of the samples were at the same level, indicating a good sample quality. 

### 2.4. Functional Annotation and Subcellular Localization of Kmal Proteins

**Figure 4:**
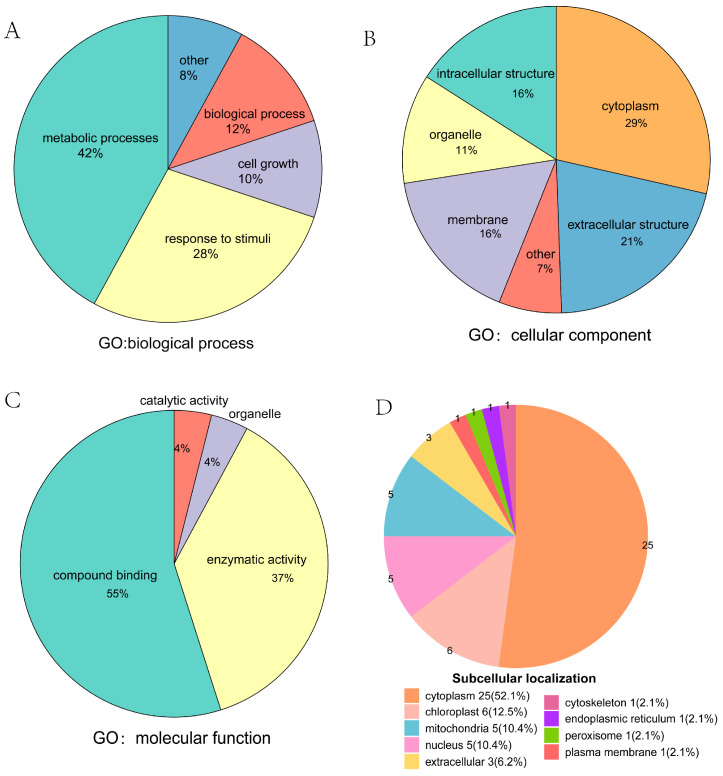
Classification of identified proteins in *P. grandiflorus*. (**A**) GO Classification of malonylated proteins in *P. grandiflorus* based on biological process. (**B**) GO classification of malonylated proteins based on cellular compartment. (**C**) GO Classification of malonylated proteins based on molecular function. (**D**) Subcellular localization of identified proteins corresponding to modified sites.

To further understand the potential roles of malonylated proteins in *P. grandiflorus*, we performed a comprehensive functional annotation of these proteins (Supplementary Figure S3A and Supplementary Table S2). Gene ontology (GO) annotation identified 409 malonylated proteins after SA treatment (Supplementary Figure S3B). In the biological processes (BP) category, 176 proteins were mainly involved in metabolic processes, including cellular and organic substance metabolism, which accounted for 42% (Figure 4A). In the cellular component (CC) category, 29% and 21% of the 182 proteins were localized in the cytoplasm and extracellular structure, respectively. (Figure 4B). Molecular function (MF) analysis of 51 proteins revealed enrichment in compound binding, representing 55% of the total (Figure 4C). Subcellular localization (Figure 4D) showed that the most malonylated proteins in *P. grandiflorus* were mainly located in the cytoplasm (52.08%), followed by the chloroplast (12.5%), mitochondria (10.42%), and nucleus (10.42%), indicating their association with cellular activities. Following SA treatment, the number of malonylated proteins in cellular components changed slightly (Supplementary Figure S3B), consistent with the observed changes in subcellular localization within the cytoplasm (Supplementary Figure S3C).

### 2.5. Functional Enrichment Analysis of Kmal Proteins

**Figure 5:**
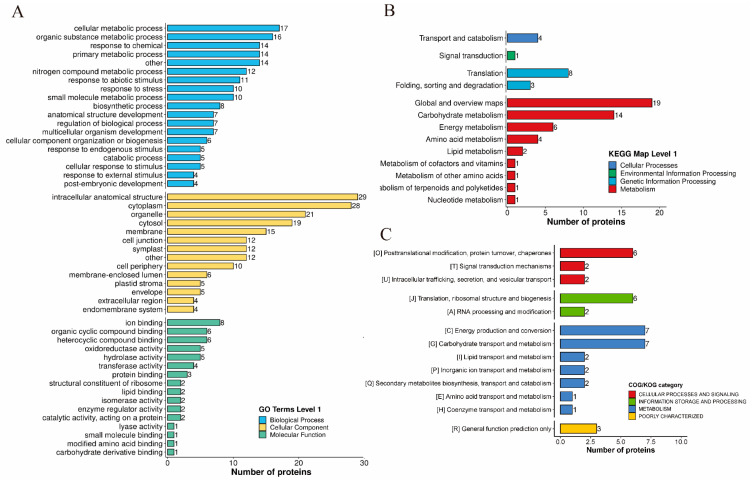
Classification of *P. grandiflorus* proteins at identification sites. (**A**) The GO classification of the malonylated proteins in *P. grandiflorus* based on biological processes, cellular components, and molecular functions. The enrichment bar graph shows the most significantly enriched functions, with the vertical axis representing the description information of the corresponding GO function, and the horizontal axis representing the enrichment significance *p* value of the log10 transformation. The larger the value, the stronger the enrichment significance. (**B**) KEGG pathway diagram of malonylation modification site. The vertical axis is the description information of the KEGG pathway, and the horizontal axis is the degree of enrichment of the differentially modified proteins after log2 conversion in this function (fold enrichment). The larger the value, the higher the degree; a blue dot color signifies stronger enrichment significance; the larger the dot, the more types of the different modified proteins. (**C**) COG/KOG pathway diagram of malonylation modification site.

Functional enrichment analysis of identified malonylated proteins was performed using the GO, the Kyoto Encylopedia of Genes and Genomes (KEGG) pathway, and Clusters of Orthologous Groups/Eukaryotic Orthologous Groups of proteins (COG/KOG) functional classifications. GO analysis revealed that the most enriched BPs, including nicotinamide nucleotide, pyruvate, and malate metabolism. In terms of MF, most proteins were enriched in mRNA 3′–UTR binding and malate dehydrogenase activity. GO annotation of cellular components revealed that most malonylated proteins were enriched in symplast, plasmodesma, and cell–cell junctions (Figure 5A). KEGG pathway analysis indicated significant involvement in carbon fixation in photosynthetic organisms and glycolysis/gluconeogenesis (Figure 5B). Additionally, functional classification of malonylated proteins using COG and KOG showed that most malonylated proteins were associated with metabolic processes, including energy production, conversion, and carbohydrate transport and metabolism (Figure 5C).

### 2.6. Protein Interaction Network of the Kmal Proteins in Roots of P. grandiflorus

**Figure 6:**
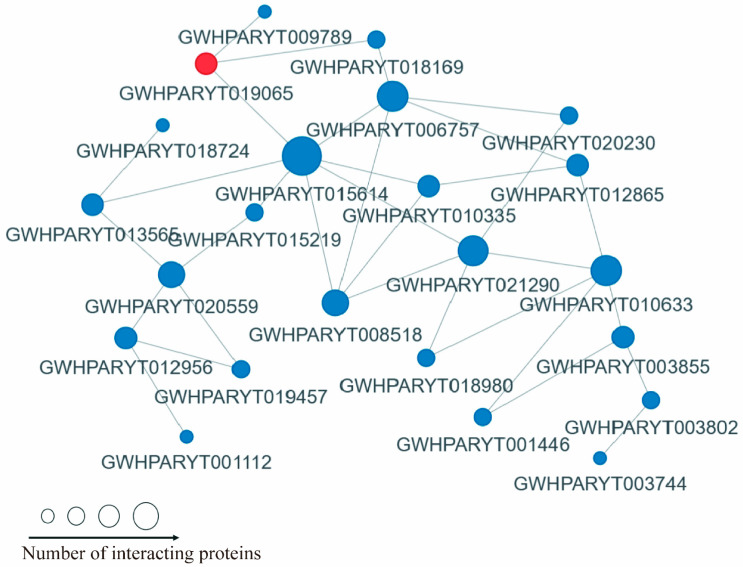
PPI relationship of malobylation proteins in *P. grandiflorus*. The blue color represents the downregulated modified proteins, and the red color represents the upregulated modified ones. The size indicates the number of proteins interacting with it.

To illustrate the interaction between proteins, we selected the top 50 proteins with the closest interactions and mapped the protein–protein interaction network (Figure 6, Supplementary Table S3). These proteins interacted with their neighbors within the network. As shown in Supplementary Table S3, almost all of these malonylated proteins are enriched in kinds of KEGG pathways. PPI mapping revealed that the malonylated proteins originated from the metabolisms, including carbohydrate metabolism and global and overview maps pathway. However, owing to the limited functional data regarding these protein–protein interactions, additional studies are necessary to validate these interactions.

### 2.7. Motifs and Secondary Structures of Lysine Malonylation Peptides

**Figure 7:**
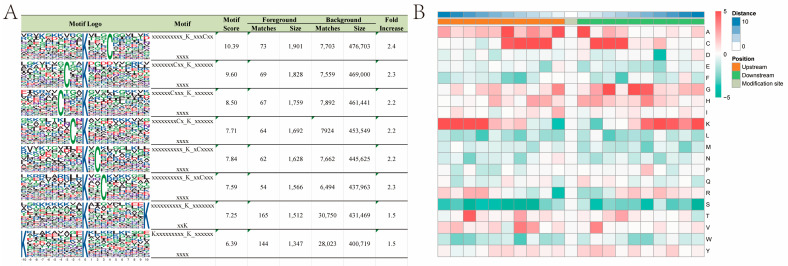
Motif analysis of the detected malonylation sites. (**A**) Sequence probability markers of the first eight enriched malonylated motifs around the malonylation sites. (**B**) A heat map of the amino acid composition around the malonylation site shows the frequencies of different types of amino acids around the residue. Red indicates enrichment and green indicates depletion.

Using Motif-x, we examined the relative frequency of amino acids surrounding K residues and identified the following eight motifs containing malonylation sites flanked by K residues: Kmal:::C, C::Kmal, C:::Kmal, C:Kmal, Kmal:C, Kmal::C, Kmal:::::::::K, and K:::::::::Kmal. The abundance of these motifs varied, with the Kmal:::C motif having the highest score among malonylated peptides (Figure 7A and Supplementary Table S4). Kmal denotes malonylated lysine, K represents lysine, and C stands for cysteine. MoMo analysis of the sequences of peptides composed of the 10 amino acids upstream and downstream of the identified malonylation sites (Figure 7B) revealed significantly enriched K residues at positions +6 to +10 and −7 to −10, while the C residues were highly enriched at positions +2 to +4 and −2 to −5. Conversely, S (serine), W (tryptophan), L (leucine), and M (methionine) residues were absent. These results suggest that malonylation in *P. grandiflorus* proteins likely occurs near lysine and cysteine residues.

### 2.8. Analysis of Differential Modification Sites of Lysine Malonylated Protein

**Figure 8:**
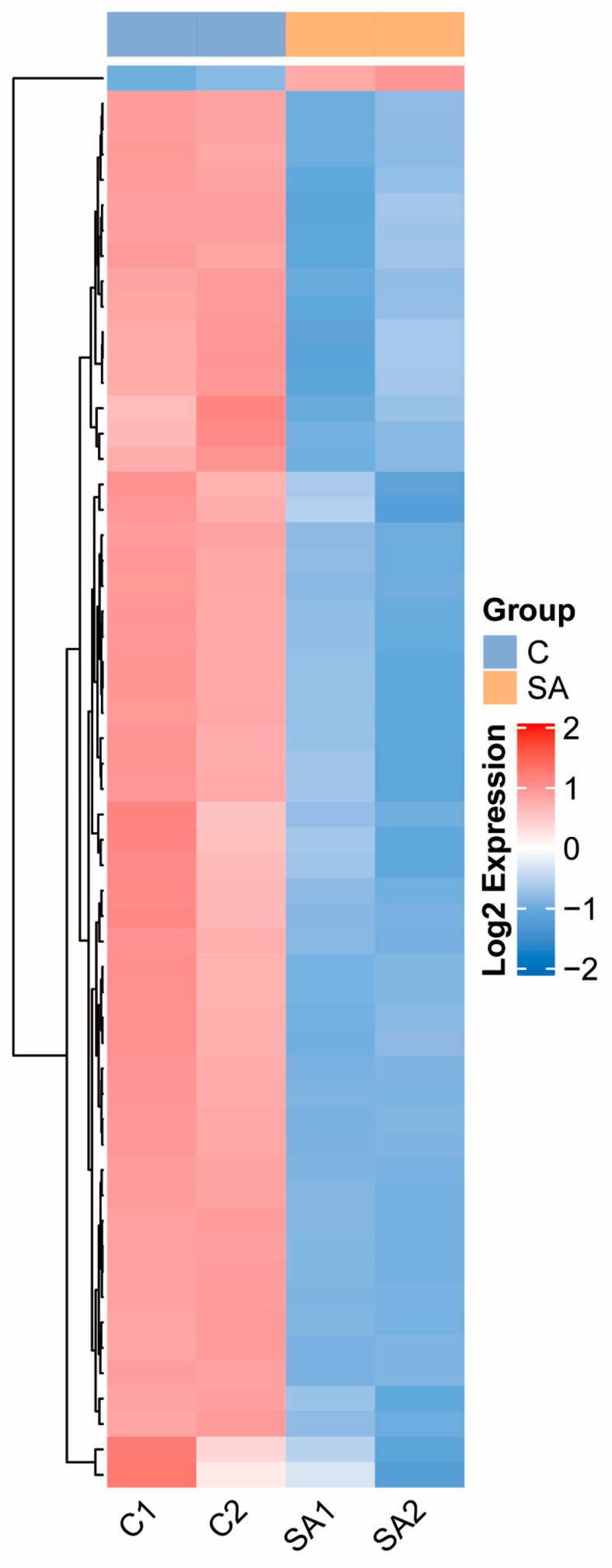
Heat map of differential modification sites. Each row is a differentially modified site, and each column is a sample. Red represents high expression, blue represents low expression, and gray represents unquantifiable in the corresponding sample.

Using a series of data processing steps and threshold selection (*p* < 0.05; coefficient of variation < 0.1), we identified differential sites and visualized their distribution across different comparison groups using bar charts. Among these differential proteins, one site in a malonylated protein was upregulated, while fifty-five sites in forty-seven malonylated proteins were downregulated (Supplementary Figure S4A and Supplementary Table S5). For comparison groups without *t*-test *p* values, scatter plots were used to display the differential distribution (Supplementary Figure S4B). Five proteins were significantly downregulated, as follows: E3N88_00970_K65, CTI12_AA280770_K109, E3N88_00970_K364, Ccrd_004295_K210, and E3N88_05445_K31. Additionally, a heatmap was constructed to display the union of differential modification sites across all comparison groups (requiring quantification in at least 2/3 of the total samples) (Figure 8). This heatmap illustrated the relative expression levels of multiple differential modification sites in *P. grandiflorus* before and after SA treatment, highlighting the clustering of relative expression levels. The relative expression levels of these differential modification sites declined to varying degrees following SA treatment. The top 30 of the most significantly different sites were selected and presented in a radar chart to illustrate their relative expression levels (Supplementary Figure S4C), revealing a complementary relationship between the expression patterns in the control and treatment groups.

### 2.9. Cluster Analysis of Differentially Expressed Lysine Malonylation Proteome

**Figure 9:**
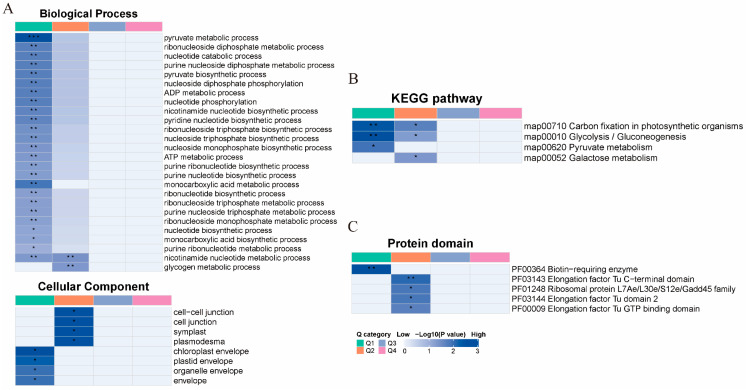
Heat map of differential proteins. (**A**) GO classification of differentially expressed proteins in different groups of different Q groups by cluster analysis. (**B**) KEGG pathways of differentially expressed proteins after cluster analysis. (**C**) Protein domains of differentially expressed proteins after cluster analysis. The color blocks corresponding to the functional descriptions of different Q groups and differentially expressed modified protein enrichment indicate the significance of enrichment. Blue indicates high significance, blue and white indicate low significance. *, **, and *** indicate *p* < 0.05, *p* < 0.01, and *p* < 0.0001, respectively.

To compare the functional similarities and differences between proteins with different fold changes, we divided them into four groups (Q1 to Q4) based on their differential expression levels. Most proteins were categorized into Q1 and Q2, with 17 modification sites in Q1, 38 in Q2, and only 1 in Q3 (Supplementary Figure S4D and Supplementary Table S6). Hierarchical clustering, based on *p* values from Fisher’s exact test obtained from the enrichment analysis, was performed to group-related functions within different Q groups. The results of GO, KEGG, and domain functional enrichment analysis results across these three Q groups were visualized in heatmaps (Figure 9A,B). GO analysis indicated that the highest enrichment score was for the pyruvate metabolism process and it had the highest enrichment score in the Q1 section of BPs (*p* < 0.0001), followed by the nicotinamide nucleotide metabolic process and glycogen metabolic processes in Q2. The most significantly enriched KEGG pathways were carbon fixation in photosynthetic organisms and galactose metabolism in Q1 (*p* < 0.01). In the protein domains, the biotin-requiring enzyme pathway had the highest scores in Q1.

## 3. Discussion

Kmal, a conserved post-translational modification using malonyl-CoA as a donor, is common in prokaryotes and eukaryotes. While significant progress has been made in understanding malonylation in microorganisms [29,30] and mammalian systems [8,14,31], research in plants, particularly medicinal plants, remains in its early stages. Despite *P. grandiflorus* being an important medicinal herb with extensive pharmacological properties, no studies have explored malonylation in this plant. In this study, we identified 1907 malonylation sites across 809 proteins in *P. grandiflorus*, with 414 proteins and 798 modification sites quantified with high confidence. In *Escherichia coli* [29], malonylated proteins were significantly enriched in pathways associated with protein translation, energy metabolism, and fatty acid biosynthesis. In cyanobacteria [32], 598 malonylation sites were identified in 339 proteins involved in carbon metabolism and photosynthesis. In human fibroblasts [8], malonylated proteins were predominantly located in the mitochondria and linked to fatty acid metabolism. The research has suggested that similar metabolic processes in plants may be regulated by malonylated proteins. In maize [33], 1722 malonylation sites were identified on 810 proteins involved in photosynthesis, ribosomes, and oxidative phosphorylation. In rice, 421 malonylation sites were identified on 247 proteins mainly involved in carbon metabolism, glycolysis/gluconeogenesis, the tricarboxylic acid cycle, and photosynthesis [15]. In wheat [15], 342 malonylation sites were identified on 233 proteins, playing crucial roles in carbon metabolism, the Calvin cycle, and amino acid biosynthesis (Table 1) [16]. Similarly, malonylated proteins in *P. grandiflorus* also play roles in carbon metabolism and photosynthesis. These findings indicate that malonylation may conservatively regulate certain proteins across microorganisms, animals, and plants despite variations in occurrence and location.

As sessile organisms, plants produce substantial amounts of reactive oxygen species (ROS) in their organelles in response to stress, leading to oxidative stress caused by excessive ROS accumulation within cells [34]. To counteract this ROS damage, plants rely on antioxidants, including antioxidant enzymes such as SOD and POD [35]. SA, acting as a signaling molecule, enhances the activities and expression of antioxidant enzymes such as SOD and POD [36,37]. MDA, a marker of lipid peroxidation, was crucial for assessing oxidative stress in plant cells. Elevated MDA levels indicate the extent of membrane lipid peroxidation and oxidative damage [38]. The SP of the plant body were mostly enzymes and proteins involved in various metabolic processes, and the increase in SP suggests that extensive protein synthesis in response to prolonged stress and is part of the plant’s adaptive response to increased oxidative stress. In our experiment, these physiological and biochemical indices showed significant changes after 15 days of SA treatment compared to pre-treatment levels, and the physiological response of *P. grandiflorus* reached a critical stage. SOD and POD activities initially decreased, reaching their lowest levels on day 10. However, by day 15, their activities had increased sharply, exceeding the control group by approximately 1.6 times (SOD) and 1.3 times (POD). 

Photosynthesis is the primary carbon-fixing reaction process on earth, essential for the biosphere energy and material supply and for oxygen production [39,40]. In plants, chloroplasts are the main sites of photosynthesis. As a plant hormone, SA is closely related to the plant’s immune system, and its exogenous application affects plant growth and development, especially photosynthesis. SA treatment enhances photosynthetic performance and stress protein induction, improving drought tolerance in wheat [41]. In ginseng leaves, SA initially increased the net photosynthetic and saponin content [42]. In this study, SA initially increased and subsequently decreased the levels of chlorophyll a, chlorophyll b, and carotenoids in *P. grandiflorus* likely by enhancing chlorophyll synthesis genes and boosting photosynthetic efficiency. CAO catalyzes the formation of chlorophyllide b, while CHLG catalyzes the synthesis of both chlorophyll a and b [29]. *CAO* and *CHLG* expression levels, as detected by RT-qPCR, were consistent with the observed changes in chlorophyll levels, confirming that SA influences the photosynthetic rate of *P. grandiflorus*. In summary, SA treatment induced a range of physiological and biochemical changes. Malonylation analysis of *P. grandiflorus* revealed that proteins related to photosynthesis, including glyceraldehyde-3-phosphate dehydrogenase, phosphoglycerate kinase, fructose-bisphosphate aldolase, malic enzyme, and malate dehydrogenase, were malonylated and downregulated after SA treatment. These findings indicate that SA may regulate the photosynthesis and metabolic functions of *P. grandiflorus* by affecting the Kmal of various enzymes and functional proteins. 

The cytoplasm of eukaryotic cells is essential for vital cellular activities, consisting of the cytoplasmic matrix, endomembrane system, cytoskeleton, and inclusions. It is crucial for cellular physiological activities. The cytoplasmic matrix is the primary site for metabolic processes, housing enzymes that catalyze various reactions [43]. In *P. grandiflorus*, most malonylated proteins (52.8%) were located in the cytoplasm, and GO analysis also revealed their predominant association with metabolic processes. Under exogenous SA treatment, metabolic activity was inhibited in the roots of *P. grandiflorus*, decreasing the protein levels in the cytoplasm. Glycolysis, the glucose-to-pyruvate conversion via the Embden–Meyerhof–Parnas pathway, generates ATP, reducing agents, and pyruvate. This process is vital for plant metabolism and respiration and supports other pathways [44,45]. The final step in the glycolysis pathway is an irreversible reaction catalyzed by pyruvate kinase [46]. Under exogenous SA stress in *P. grandiflorus*, seven differentially expressed malonylated proteins related to the glycolysis process were identified (Supplementary Table S7). Pyruvate and acetyl-CoA produced by the glycolytic pathway are precursors of terpenoid compounds. These precursors enter the mevalonate (MVA) and methylerythritol phosphate (MEP) pathways, which are involved in the biosynthesis of platycodin saponins [47]. Cytochrome P450 monooxygenases (CYP450s) are enzymes in the biosynthetic pathway of triterpenoid saponins. Together with enzymes such as oxidosqualene cyclases and uridine diphosphate glycosyltransferases, they catalyze the formation of various triterpenoid saponins with different main chains from 2,3-oxidosqualene [48]. In our experiments, we identified a Kmal modification at one site of CYP72A555. This suggests that salicylic acid (SA) may influence the glycolytic pathway proteins and malonylation of CYP450s, thereby affecting the biosynthesis of platycodin saponins.

Phospholipase D (PLD), a member of the phospholipase superfamily, catalyzes the hydrolysis of phosphatidylcholine to phosphatidic acid (PA) [49]. In plants, PLD is closely associated with growth, development, stress responses, and signal transduction. It is activated under stress conditions, such as drought, salinity, and pathogen infection, and enhances the plant’s ability to adapt to environmental changes [50]. Phospholipid signaling plays a crucial role as a regulatory lipid in plant cellular stress responses [51]. Research has shown that PLD is a critical component of the salicylic acid (SA) signaling pathway. Krinke et al. [52] showed that SA application rapidly activates PLD, establishing its role within the SA signaling cascade [53]. PLDα1, a subtype of PLD, has also been implicated in signal transduction [52]. In SA-treated *P. grandiflorus*, both PLD and PLDα1 were found to undergo malonylation. Notably, PLDα1 was downregulated and involved in the KEGG glycerophospholipid and ether lipid pathways. These findings provide new research directions for elucidating plant signaling mechanisms and adaptive strategies to environmental stresses.

## 4. Materials and Methods

### 4.1. Plants and SA Treatment

The *P. grandiflorus* plant material used in this study has been described by Su et al. [54]. The specimens were preserved in the specimen room of Anhui University of Chinese Medicine (number of 20,200,705). Four similarly grown *P. grandiflorus* plants were selected and divided into two groups. SA solution (1.0 g/L) was sprayed on the leaves of plants in the experimental group at 9:00 a.m. and 6:00 p.m. daily, while distilled water was sprayed on the leaves of plants in the control group until water dripped from both sides of the leaves. Treatment continued for 15 days.

### 4.2. Physiological and Biochemical Detection

Fresh leaves of *P. grandiflorus* were collected on days 0, 5, 10, and 15 of SA treatment to detect physiological and biochemical indices (three biological replicates per group). The MDA level was measured using the thiobarbituric acid (TBA) method, as described by Velikova et al. [55]. SOD activity was determined using the nitro blue tetrazolium chloride method [56]. POD activity was quantified using the guaiacol method [57]. SP content was calculated using the Coomassie Brilliant Blue method [58], while chlorophyll content in the leaves was measured using the 95% ethanol grinding–filtration method [59].

### 4.3. Determination of Total Saponin Content

Root samples of *P. grandiflorus* were collected at the above four time points, dried, and ground into powder. The total saponin content was determined using a modified version of the method described by Sun et al. [60]. Specifically, 0.5 g of the powder was mixed with 10 mL of 70% methanol, subjected to ultrasound at 80 W for 30 min, and centrifuged at 2660× *g* for 20 min. An aliquot of 200 μL of supernatant was taken, mixed with 0.2 mL of 5% vanillin–glacial acetic acid solution and 0.8 mL of perchloric acid, and incubated in a 60 °C water bath for 15 min. After cooling, 4 mL of glacial acetic acid was added, and the absorbance was measured at 472 nm.

### 4.4. Protein Extraction and Western Blot Analysis

Roots from the experimental and control groups were collected as samples and washed with ddH_2_O. After pre-cooling the mortar with liquid nitrogen, an appropriate amount of root was added into liquid nitrogen, ground into powder, and transferred to a new centrifuge tube. Four volumes of 10% TCA/acetone were added to each group and allowed to stand for 4 h (−20 °C). Samples were centrifuged at 4 °C for 5 min (4500× *g*), the supernatant was discarded, and the pellet was washed thrice with pre-cooled acetone and air dried. The pellet was then lysed with 4 volumes of SDS lysis buffer (containing 1% SDS, 1% protease inhibitor, 3 μM TSA, and 50 mM NAM), sonicated, and lysed. After adding an equal volume of Tris-balanced phenol, samples were centrifuged for 10 min (4 °C, 5500× *g*). The upper phenol phase was transferred to a new centrifuge tube and precipitated overnight with five volumes of 0.1 M ammonium acetate/methanol. The precipitate was then washed with methanol and acetone, reconstituted with 8 M urea, and the resulting protein concentration was determined using the BCA kit (Beyotime Biotechnology, Shanghai, China).

For Western blot analysis, the eluted target proteins (20 mg) were separated using 12% sodium lauryl sulfate–polyacrylamide gel electrophoresis, and electrophoresis was performed on a polyvinylidene fluoride membrane. Anti-malonyl-lysine antibodies (PTM-902; Lot: 23056103M119, 1:1000 dilution, PTM Biolabs, Hangzhou, China) were used to detect malonylated proteins, followed by secondary antibodies against mouse immunoglobulin G (IgG) (31430, 1:1000 dilution, Thermo Fisher Scientific, Waltham, MA, USA).

### 4.5. Protein Trypsin Digestion

An equal amount of each protein sample was enzymatically hydrolyzed by adjusting its volume by adding lysis solution, slowly adding tricarboxylic acid to a final concentration of 20%, and mixing. The samples were precipitated for 2 h (4 °C), centrifuged for 5 min (4500× *g*), the supernatant discarded, and the resulting precipitate was washed with precooled acetone 2–3 times. After drying, TEAB was added at a final concentration of 200 mM, sonicated to mix the precipitate, and an appropriate amount of trypsin was added at a ratio of 1:50 (protease/protein, m/m), followed by hydrolysis overnight. Dithiothreitol was added at a final concentration of 5 mM and reduced at 56 °C for 30 min. Finally, iodoacetamide was added to a final concentration of 11 mM and incubated for 15 min at room temperature in the dark.

### 4.6. Affinity Enrichment of Modified Peptides

The peptides were dissolved in NETN buffer (100 mM NaCl, 1 mM EDTA, 50 mM Tris-HCl, 0.5% NP-40, pH of 8.0), and the resulting supernatant was transferred to the pre-washed antibody beads (lot number PTM 904, PTM Biolabs, Hangzhou, China), placed on a rotating shaker at 4 °C, gently shaken, and incubated overnight. Following that, the beads were washed four times with NETN buffer and twice with H_2_O. The bound peptides were eluted from the beads using 0.1% trifluoroacetic acid. Finally, the eluted fractions were combined and vacuum-dried. For LC-MS/MS analysis, the resulting peptides were desalted with C18 ZipTips (Millipore, Burlington, MA, USA) following the manufacturer’s instructions.

### 4.7. Quantitative Proteomic Analysis by LC-MS/MS and Mass-Spectrum Quality Control Analysis

The peptides were first dissolved in mobile phase A of the liquid chromatography system, before being separated using the Easy-nLC1000 (Thermo Scientific, Waltham, MA, USA) ultra-high-performance liquid phase system. Mobile phase A is an aqueous solution containing 0.1% formic acid and 2% acetonitrile, while mobile phase B contains 90% acetonitrile and 0.1% formic acid. The elution gradient was set as follows: 0–42 min, 9–25% B; 42–52 min, 25–35% B; 52–56 min, 35–90% B; 56–60 min, 90% B, at a constant flow rate of 450 nL/min. The separated peptides were then ionized using a capillary ion and then analyzed by timsTOF Pro mass spectrometry. The ion source voltage was set to 1.65 kV, with both the peptide parent ion and its secondary fragments are detected and analyzed using high-resolution TOF. The scanning range of the secondary mass spectrometry was set to 100–1700. Data were collected using parallel cumulative serial fragmentation (PASEF) mode. After primary mass spectrometry, 10 PASEF modes were performed to capture secondary spectra for ions with charge states of 0–5. The dynamic exclusion time of the series mass spectrometry scan was set to 24 s to avoid repeated scanning of the same ion.

### 4.8. Database Search, Quantitative Analysis and Differential Modification Site Screening

Data from LC-MS/MS were retrieved using the MaxQuant (v1.6.15.0) search engine [61]. The database used was Blast_Platycodon_grandiflorus_94286_GWH_20230727.fasta (21,619 sequences). Reverse decoy was added to calculate the false positive rate (FDR) because of random matching, and a common contamination library was added to the database to eliminate the influence of contaminating proteins in the identification results. Trypsin/P was used for enzyme digestion. The number of missing cut sites was set to 4, the minimum peptide length was set at 7 amino acid residues, the maximum number of peptide modifications was set to 5, the mass tolerance for precursor ions was set as 20 ppm in first search and 20 ppm in main search, respectively, and the mass tolerance for secondary fragment ions was 20 ppm. Carbamidomethylation of cysteine was set as a fixed modification, while variable modifications included methionine oxidation, protein N-terminus acetylation, and Kmal. The FDR for protein and peptide-spectrum match identification was set to 1%. To ensure high-quality results, further filtering was applied, with the following filtering conditions: precursor and protein FDR were set to 1%. Identified proteins must contain at least one unique peptide.

For the relative quantification of each modified peptides, the original LC-MS/MS dataset was processed against the database and converted into a matrix of peptide intensities. These intensities (*I*) were centralized and transformed into relative quantitative values (*R*) using the formula below, where *i* denotes the sample and *j* denotes the modified peptide.

Rij=Iij/Mean

When proteomic and PTM analyses were performed on the same group, the relative quantification values of the modified peptides were divided by the relative quantification values of the corresponding proteins. This step helps to eliminate the effects of modified protein expression.

The ratio of the mean relative quantification values of the modification sites in the two groups of samples is given as fold change (*FC*). Repeat triplicates to calculate the mean. The formula is as follows, where k is the modification site.

*FC_A_*_/*B*,*k*_ = *Mean* (*R_ik_*, *i* ∈ *A*)/*Mean* (*R_ik_*, *i* ∈ *B*)

To assess the significant differences, the standard coefficient of variation (*CV*) of each modified site in the group was used as the index, and the default *CV* was <0.1. The formula is as follows:

*CV_k_* = *SD* (*A*_1_*_k_*/*B*_1_*_k_*, *A*_2_*_k_*/*B*_2_*_k_*)/*Mean* (*A*_1*k*_/*B*_1_*_k_*, *A*_2_*_k_*/*B*_2_*_k_*)

If the ratio of differential expression is greater than 1.5, it is treated as being significantly upregulated; meanwhile, when the ratio is less than 1/1.5, it is considered as downregulated.

### 4.9. Protein Annotation and Enrichment Analysis

The identified proteins were analyzed using eggnog-mapper software based on the eggNOG database (v5.0.2, http://eggnog5.embl.de/#/app/home, accessed on 20 September 2023) to extract the GO IDs for functional classification of the proteins according to cell composition, MF, and BP [62]. Protein domains were annotated with PfamScan tools using the pfam database (A.hmm-33.1, https://www.ebi.ac.uk/interpro/entry/pfam/#table, accessed on 19 September 2023) [63]. The KEGG pathway database was used to annotate protein pathways through BLAST (blastp, evalue ≤ 1 × 10^−4^) with the highest scores [64,65]. The WoLF PSORT software (v0.2) was used to predict the subcellular localization of submitted proteins [66]. The eggnog-mapper software, eggNOG (v5.0.2, http://eggnog5.embl.de/#/app/home, accessed on 18 September 2023), and NCBI (COG2020, KOG, https://ftp.ncbi.nih.gov/pub/COG/, accessed on 20 September 2023) were used to annotate the database with COG [67]. Fisher’s exact test was used for the enrichment analysis of differentially expressed modified proteins, with a *p* value < 0.05 considered as statistically significant [68]. 

### 4.10. Motif Analysis and Protein–Protein Interaction Analysis

The sequence characteristics of the modification sites were analyzed using the MoMo analysis tool (v 5.5.4) [69] based on the motif-x algorithm. Peptide sequences consisting of 10 amino acids upstream and downstream of the identified modification sites were used. The background was set to sequences of 10 residues surrounding all potential modification sites. A characteristic sequence was defined when it contained over 20 peptides and had a *p* value less than 0.000001. 

Differential proteins with a fold change of ≥1.5 were selected and compared against the STRING protein interaction network database, extracting interactions with a confidence score > 0.7 (high confidence). The R package “visNetwork” tool (v 2.1.2) was used to visualize the differential protein interaction network.

### 4.11. Quantitative Real-Time PCR

Total RNA was extracted from powdered samples using TRIzol reagent and reversed transcribed into cDNA using the Evo M-MLV RT Premix for qPCR kit (TaKaRa, Osaka, Japan) according to the manufacturer’s instructions. Quantitative real-time PCR (RT-qPCR) was performed with a real-time fluorescence quantitative PCR instrument using the SYBR Green Pro Taq HS premixed qPCR kit (Accurate Biology, Changsha, China). The 18S gene was used as an internal reference, and the primers used for RT-qPCR are listed in Supplementary Table S8. The relative expression levels of each gene were calculated using the 2^−ΔΔCt^ method based on the Ct values obtained from the RT-qPCR instrument. Each sample was analyzed in triplicate.

## 5. Conclusions

In this study, it was demonstrated that SA significantly influenced the physiological and metabolic processes of *P. grandiflorus*. Salicylic acid can affect the levels of malonylated proteins, especially the expression of photosynthetic and glycolytic enzymes. In addition, PLD, PLDα1, and cytochrome P450s of the triterpene saponin synthesis pathway in Platycodon grandiflorum were malonylated. These findings highlight the complex interactions between metabolic pathways, protein modifications, and stress responses, providing new insights into plant signaling mechanisms and their adaptive strategies to environmental changes. These findings enhance our understanding of plant stress response mechanisms and may optimize the utilization of medicinal plants. 

## Figures and Tables

**Table 1:** Comparison of the number of malonylated proteins and sites in *P. grandiflorus* with previous studies in other species.

Species	Kmal Proteins	Kmal Sites	References
*Escherichia coli*	594	1745	Qian et al., 2016 [29]
*Cyanobacteria*	339	598	Ma et al., 2017 [32]
Maize (*Zea mays* L.)	810	1722	Xu et al., 2021 [33]
Rice (*Oryza sativa)*	247	421	Mujahid et al., 2017 [15]
Wheat	233	342	Liu et al., 2018 [16]
*Bacillus amylolique faciens* FZB42	382	809	Fan et al., 2017 [12]
*P. grandifloru*s	809	1907	

## Data Availability

The original data presented in this study are openly available in [PRIDE] at https://www.ebi.ac.uk/pride/archive/ (accessed on 9 December 2024) with the dataset identifier PXD058676.

## Supplementary Materials

The following supporting information can be downloaded at: https://www.mdpi.com/article/10.3390/ijms26031392/s1.
