# Supplementary material for: Quantitative Proteomic Analysis of Lysine Malonylation in Response to Salicylic Acid in the Roots of Platycodon grandiflorus
Source: Int J Mol Sci. 2025 Feb 6;26(3):1392. doi: 10.3390/ijms26031392 (PMC11818218; doi:10.3390/ijms26031392)
Supplement: Supplementary file 1 [file ijms-26-01392-s001.zip › Supplementary Figures S1-S4.pdf]

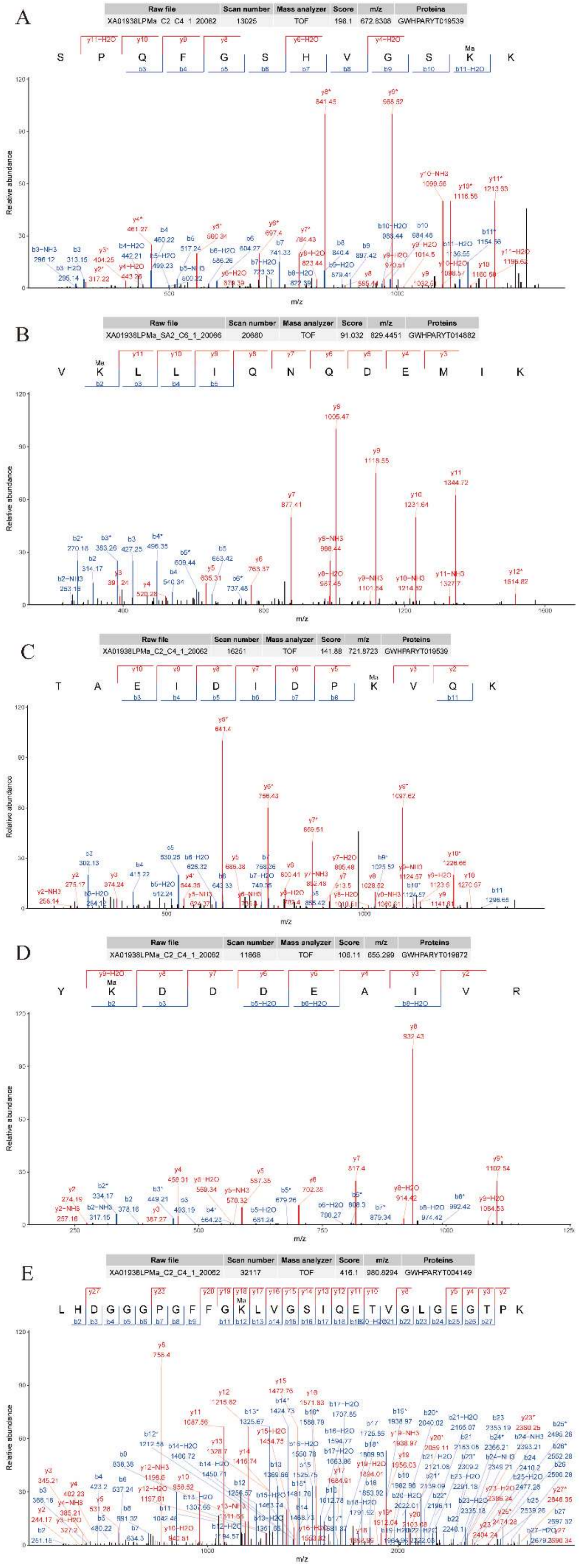

Supplementary Figure S1. Representative MS/MS spectra of malonylated from five peptides. (**A**) Malonylated peptide at site E3N88_00970_K65 SPQFGSHVGSKmalK. (**B**) Malonylated peptide at site

CTI12_AA280770_K109 VKmalLLIQNQDEMIK. (**C**) Malonylated peptide at site E3N88_00970_K364 TAEIDIDPKmalVQK. (**D**) Malonylated peptide at site Ccrd_004295_K210 YKmalDDDEAIVR. (**E**) Malonylated peptide at site E3N88_05445_K31 LHDGGGPGFFGKmalLVGSIQETVGLGEGTPK.

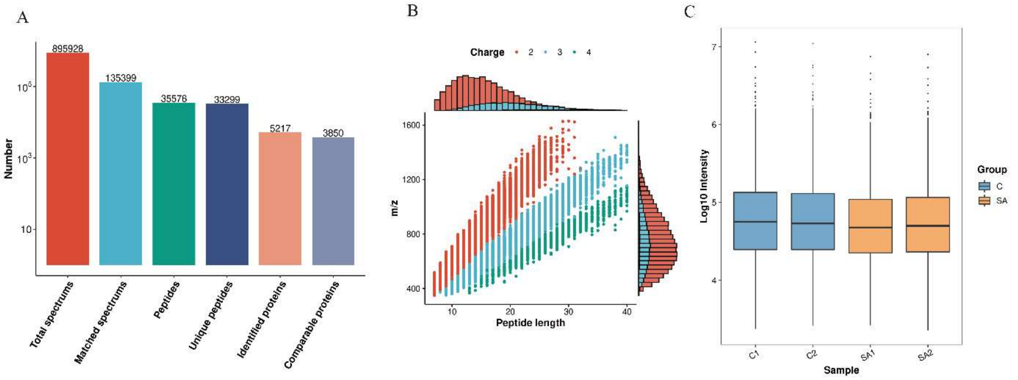

Supplementary Figure S2. The basic characteristics of malonylated proteins identified in *P. grandiflorus.* (**A**) The overall number of peptides and proteins identified. (**B**) The peptide length distribution. (**C**) A box diagram of the intensity value of the modified site. The mean of the sample is on the same horizontal line, indicating that the sample is of good quality.

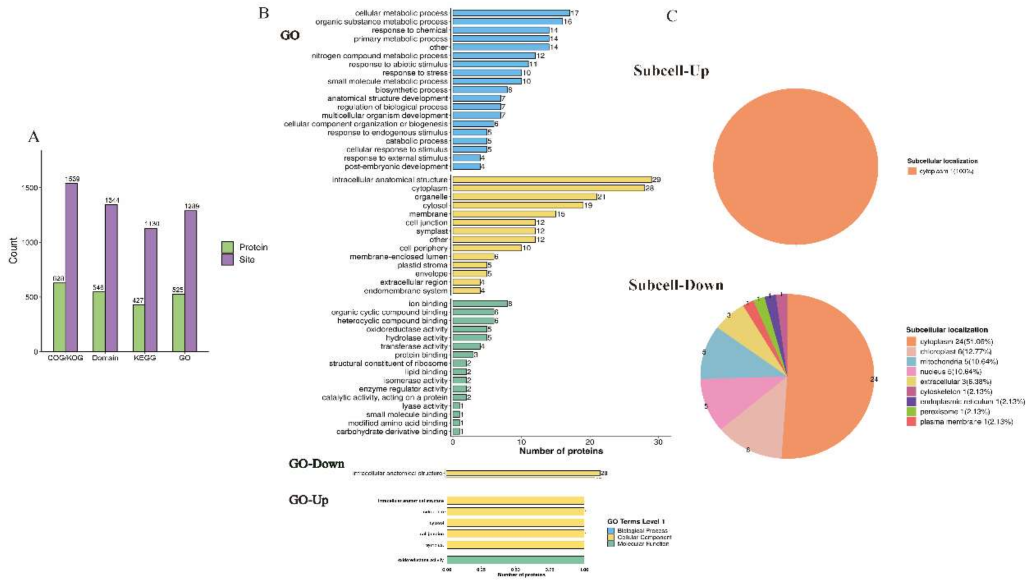

Supplementary Figure S3. Protein function annotation if identified proteins in *P. grandiflorus.* (**A**) Classification of identified proteins and modified sites (**B**) GO enrichment analysis of differential proteins and sites. (**C**) Subcellular localization of differential proteins.

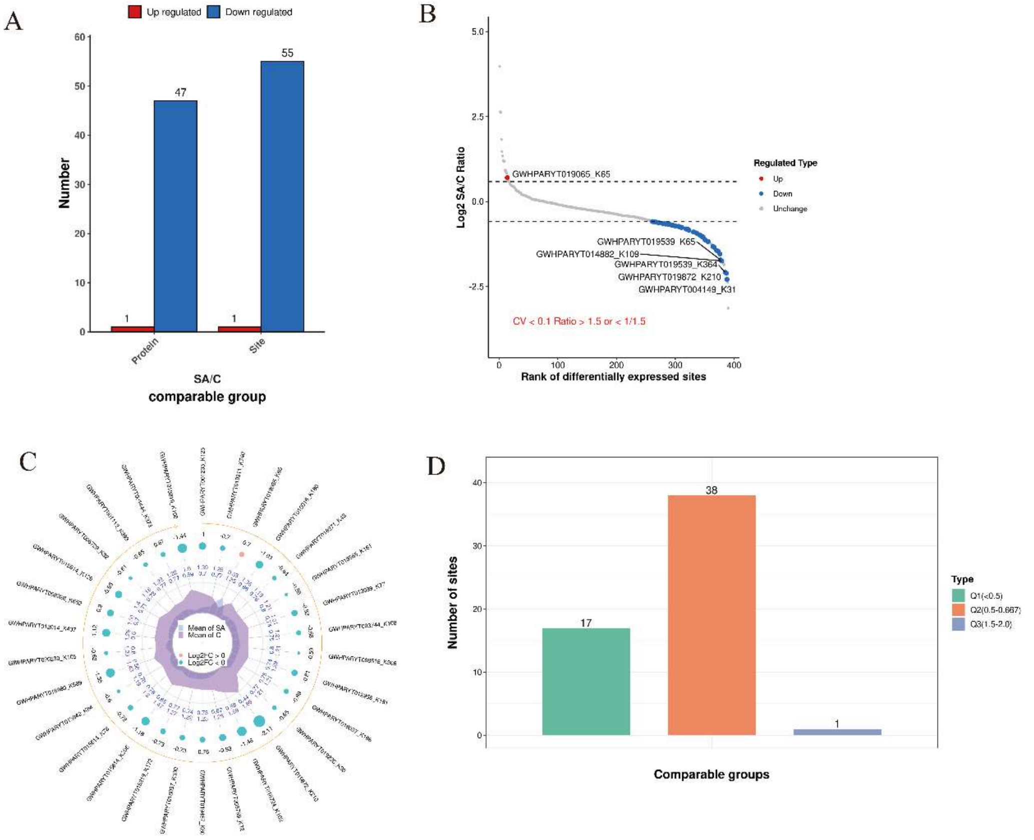

Supplementary Figure S4. Statistical Plot of Differentially Modified Sites. (**A**) A scatter plot of differential modification sites. The red dots indicate that the significant difference is upregulated, the blue dots indicate that the significant difference is downregulated, and the gray indicates that there is no significant difference. (**B**) A heat map of differential modification sites. Red represents high expression, blue represents low expression, and gray indicates that it is not quantifiable in the corresponding sample. (**C**) A radar map of differential modification sites. Pink indicates upregulation and light blue indicates downregulation. The larger the point, the greater the multiple of the difference, and the spike indicates that the expression of differential modification sites is high. (**D**) The number of sites in different groups.
